# Methylene Blue Alleviates Thiamethoxam-Induced Toxicity in Honeybee Larvae by Activating Dihydrolipoyl Dehydrogenase

**DOI:** 10.3390/insects17030334

**Published:** 2026-03-19

**Authors:** Xiao-Shi He, Jia-Wei Huang, Chang-Hao Chu, Qi-Bao He, Min Liao, Lin-Sheng Yu, Ping-Li Dai, Yong Huang, Hai-Qun Cao

**Affiliations:** 1 Institute of Apicultural Research, School of Plant Protection, Anhui Agricultural University, Hefei 230036, China; he1431745459@163.com (X.-S.H.); hjw1429092226@163.com (J.-W.H.); 17864066525@163.com (C.-H.C.); heqibao0418@126.com (Q.-B.H.); liaomin3119@126.com (M.L.); yulinsheng@ahau.edu.cn (L.-S.Y.); 2 Key Laboratory of Agro-Products Quality and Biosafety, Anhui Agricultural University, Ministry of Education, Hefei 230036, China; 3 State Key Laboratory of Resource Insects, Institute of Apicultural Research, Chinese Academy of Agricultural Sciences, Beijing 100193, China; daipingli@caas.cn

**Keywords:** methylene blue, dihydrolipoamide dehydrogenase, *Apis mellifera*, RNA interference, toxicology

## Abstract

The extensive utilization of TMX, a substance characterized by its high toxicity towards honeybees, has exerted a deleterious influence on the employment of neonicotinoid insecticides and the proliferation of bee colonies. However, there is a lack of effective solutions to mitigate the toxicological impact of neonicotinoid insecticides on bees. The present study proposes a method of using MB to alleviate TMX poisoning in honeybee (*Apis mellifera ligustica)* larvae. The results demonstrated that when bee larvae ingested MB at a concentration of 0.32 mg·L^−1^, the mortality rate of larvae could be reduced from 47.2% to 25.0%. Transcriptome analysis identified the honeybee dihydrolipoyl dehydrogenase (*AmDld*) gene as one of the main genes involved in the function of MB. *AmDld* was highly expressed in larval hemolymph. Its expression levels and enzymatic content were suppressed by either TMX or MB alone but restored by the TMX+MB combination. RNAi-mediated knockdown of *AmDld* decreased AmDld content and increased larval mortality under the TMX+MB co-treatment from 25.0% to 40.6%. This indicated that the TMX+MB combination rescued *AmDld* levels, thereby alleviating TMX toxicity to bee larvae. The present study has demonstrated that the ingestion of MB by honeybee larvae has the capacity to reduce the toxicity of TMX, a toxic substance, through the action of the *AmDld* gene. This provides a novel approach to mitigating pesticide poisoning in bees.

## Simple Summary

The extensive use of thiamethoxam (TMX) pesticides has led to a significant decline in bee populations, causing irreversible damage to the sustainable development of bee colonies and attracting widespread global attention. TMX, a neonicotinoid insecticide that has gained significant popularity, has been observed to exert a deleterious effect on bee development. However, there is currently a lack of effective protective agents against TMX poisoning in bees. Preliminary laboratory experiments have identified methylene blue (MB) as a substance capable of protecting bee larvae against TMX poisoning. In this study, we utilized a multifaceted approach encompassing bioassays, transcriptome sequencing, RNA interference (RNAi), and enzyme content assays. This comprehensive strategy enabled us to demonstrate the detoxification efficacy of MB, identify target genes, and validate their functions. This provides a novel experimental approach for developing protective agents against pesticide poisoning in bees.

## 1. Introduction

It is an irrefutable fact that honeybees play an integral role in the maintenance of global food security, biodiversity, and economic stability. They are vital pollinators of many plant crops and producers of commercially important products such as honey and wax [1]. Recent years have seen considerable attention focused on the effects of pesticides, especially neonicotinoids, on bees [2]. Neonicotinoid insecticides are a class of neurotoxic insecticides that are widely used in contemporary agricultural systems due to their extensive spectrum of pest control efficacy and systemic uptake by plants [3].

TMX is a key representative of neonicotinoid insecticides, which are widely used on crops that produce nectar and are rich in pollen [4,5]. A growing literature has documented the multifaceted adverse effects of TMX exposure on bee physiology and behaviour. For instance, sublethal concentrations of TMX can disrupt the circadian rhythm regulation of *Apis mellifera* and *Bombus terrestris*. This leads to changes in the time windows and reduced foraging efficiency [6]. Furthermore, non-lethal exposure of honeybees to TMX results in high mortality rates, as the bees are unable to return to the hive. This level of exposure is sufficient to place entire colonies at risk of collapse [7]. A substantial corpus of research has demonstrated that chronic exposure to TMX at the colony level is associated with a decline in brood survival rates, a reduction in the lifespan of worker bees, and an elevated incidence of queen supersedure [8].

MB has attracted considerable attention in the fields of toxicology and pharmacology in recent years, largely due to the discovery of its diverse and novel biological activities. It has been demonstrated that MB exerts a dose-dependent reduction in the neurotoxicity of cyanide, thereby preventing lethal inhibition of respiratory medullary neurons and fatal outcomes. The substance is effective in treating ifosfamide-induced neurotoxicity and refractory vasomotor shock. Most notably, it has been used as an antidote for acquired methemoglobinemia (MetHb) [9,10]. Furthermore, this substance has been found to have antioxidant, anti-inflammatory, and neuroprotective properties. Furthermore, it has been demonstrated that this substance can modulate the cellular redox status, thereby mitigating damage induced by various toxicants [11,12,13,14,15].

These characteristics have generated interest in its potential application for alleviating pesticide-induced toxicity in non-target organisms, such as bees. However, the efficacy of MB in alleviating TMX-induced poisoning in honeybees remains unknown. This study investigated the effects and mechanisms of MB on the toxicity of TMX to honeybee larvae, given its significant potential as a targeted detoxifier for bee pesticide poisoning. This was achieved through virulence assays, transcriptomics, RNAi, and enzyme content measurements. This research aims to elucidate the molecular mechanisms of MB-mediated detoxification as well as the application of MB in mitigating TMX poisoning in bees.

## 2. Materials and Methods

### 2.1. Insects

The bee colonies were collected from the apiary of the School of Plant Protection at Anhui Agricultural University in Hefei, Anhui Province, China. The experiment was conducted between July 2024 and August 2025. During this period, the bee colonies were healthy without exposure to any pesticides.

### 2.2. Chemicals and Reagents

TMX (purity 97%) was purchased from Anhui Guangxin Agrochemical Co., Ltd. (Xuancheng, China), and MB was from Shanghai Macklin Biochemical Co., Ltd. (Shanghai, China). Yeast powder was derived from biotopped: YM6050D, D-glucose from biotopped: G6150D, and D-fructose from biotopped: F6100D. T7 RiboMAX™ Express Large Scale RNA Production System was purchased from Promega (Beijing) Biotech Co., Ltd. (Beijing, China). Commercial *Dld* Enzyme-Linked Immunosorbent Assay (ELISA) Kit purchased from Yuanju Bio (Shanghai, China). The fresh royal jelly used in the experiment was provided by the apiaries of the School of Plant Protection at Anhui Agricultural University.

### 2.3. Honeybee Larvae-Rearing Conditions

The preparation of the basic diet (Bd) for honeybee larvae involves the utilization of fresh royal jelly, D-fructose, D-glucose, sterile water, and yeast powder in a mass ratio of 50:6:6:37:1 (m/m) [16]. Three days before the commencement of the experiment, the queen excluder must be inserted into the designated experimental colony, with an empty comb placed between the excluder frames. Subsequent to a period of three days, during which the eggs hatched into larvae, the comb was removed. The 1-day-old larvae were collected and distributed into the wells of a 48-well culture plate, containing the Bd (200 μL per well) [17]. The plate was then placed in a culture chamber, where it was maintained at 34 ± 1 °C, in conditions of 70 ± 5% relative humidity and in the absence of light [18]. After a two-day adaptation period, any larvae showing signs of damage, disease or dying due to bacterial infection will be removed, and the healthy larvae will be randomly grouped to begin the feeding experiment.

### 2.4. Detoxification Effect Measurement

Transfer 1-day-old larvae into 48-well culture plates, with one larva in each well. Then, randomly divide the healthy 3-day-old larvae into six groups. The experimental treatments include the following: Control, MB, TMX, TMX + MB-C1, TMX + MB-C2, and TMX + MB-C3. Three duplicate samples were set for each group, and 24 larvae were used for each duplicate sample. On the third day, the control group was provided with a standard Bd, the MB group was fed a diet containing 1.6 mg·L^−1^ of MB, and the other groups were fed a diet containing 40 mg·L^−1^ TMX. On the fourth day, the control group was administered Bd, the group MB was fed a diet containing 1.6 mg·L^−1^ of MB, and the TMX group was given a diet containing 40 mg·L^−1^ TMX. The TMX + MB-C1, -C2 and -C3 groups were co-administered MB at concentrations of 0.064, 0.32 and 1.6 mg·L^−1^, respectively. On the fifth day, the larval mortality rate of each group was calculated.

### 2.5. Sample Preparation

**Figure 1:**
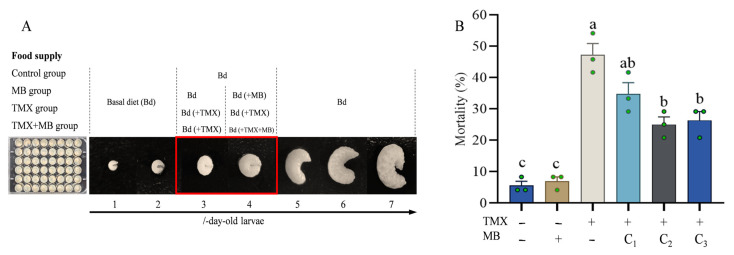
The alleviating effect of MB on TMX. (**A**) Schematic of honeybee larval culture and treatment. (**B**) The alleviating effect of MB at different concentrations. Data were analyzed using the one-way analysis of variance (ANOVA) with Tukey’s multiple comparisons test, and Student’s *t* test were employed; bars represent mean ± SD (*n* = 3, *p* < 0.001). Differences between groups are denoted by letters, with *p* < 0.05 representing the threshold for statistical significance. TMX: thiamethoxam, MB: methylene blue. “+” indicates addition, “−” indicates no addition. The red box represents the main processing period. The green dots indicate repetition.

Healthy 3-day-old larvae were randomly assigned to four experimental groups, with five biological replicates per group. On the third day, the Control group and the MB group were administered Bd, whereas the TMX and TMX+MB groups were fed with Bd containing 40 mg·L^−1^ TMX. On the fourth day, the dietary regimens were adjusted: the Control and TMX groups continued their respective diets, while the MB and TMX + MB groups were switched to diets containing 0.32 mg·L^−1^ MB and 40 mg·L^−1^ TMX plus 0.32 mg·L^−1^ MB, respectively. On the fifth day, larvae were collected as samples for RNA extraction (Figure 1A).

Concurrently, additional larvae were provided with Bd for tissue sampling. 5-day-old larvae were dissected in Phosphate-Buffered Saline (PBS) to obtain four distinct tissues, namely the integument, fat body, hemolymph and midgut. The collection of samples was conducted with five replicates.

### 2.6. RNA Extraction and cDNA Synthesis

Individual larval samples were transferred to 1.5 mL RNase-free centrifuge tubes. For each tube, an appropriate volume of Trizol reagent and two RNase-free steel beads (3 mm in diameter) were added. The tubes were then placed in a tissue grinder, and larvae were homogenized at 60 Hz for 2 min to ensure complete cell lysis. The total RNA was extracted in strict accordance with the manufacturer’s protocol. The extracted RNA was then immediately stored at −80 °C to prevent degradation. The concentration and purity of the total RNA were then quantified using a DS-11 Spectrophotometer (DeNovix, Wilmington, DE, USA), with the absorbance ratios (A_260_/A_280_ and A_260_/A_230_) used to assess RNA purity (optimal range: A_260_/A_280_ = 1.8–2.0, A_260_/A_230_ > 1.8). Furthermore, the integrity of RNA was determined by subjecting the samples to 1.0% agarose gel electrophoresis, with 4S green plus used for staining. The presence of clear, sharp 28S and 18S rRNA bands (with a brightness ratio of approximately 2:1) was indicative of high-quality RNA. Finally, first-strand complementary DNA (cDNA) was synthesized from qualified total RNA using a commercial reverse transcription kit (Vazyme HiScript^®^lll RT SuperMix for qPCR (+gDNA wiper)). The kit’s instructions were followed to ensure efficient and specific cDNA synthesis.

### 2.7. Library Preparation and Sequencing

The mRNA enrichment process was conducted on the total RNA sample using oligo (dT)-attached magnetic beads. The enriched mRNA with poly (A) tails was fragmented using a fragmentation buffer. This was followed by reverse transcription using random N6 primers to synthesize double-stranded cDNA. This double-stranded DNA was then end-repaired and 5′-phosphorylated to form a blunt end with a protruding ‘A’, followed by ligation of a bubble-shaped adapter with a protruding ‘T’. These ligation products were then subjected to PCR amplification using primers designed. The PCR products were denatured into single strands, and single-stranded circular DNA libraries were generated using a bridged primer. The constructed libraries were then subjected to rigorous quality control checks, and those that passed were sequenced. This sequencing data, also referred to as ‘raw reads’ or ‘raw data’, was then subjected to a quality control (QC) procedure. The study conducted transcriptome sequencing on 12 bee (*Apis mellifera*) samples using the DNBSEQ platform (https://www.mgi-tech.com/DNBSEQ-Technology.html, accessed on 10 December 2024), with a sequencing read length of PE150 and a quality system of Phred+33. After QC, the clean reads were aligned to the reference sequences (*Apis*_*mellifera*_7460.NCBI.GCF_003254395.2_Amel_HAv3.1.v2201). The alignment results were then subjected to a second QC to evaluate the quality of the alignment, including the alignment rate and the distribution of reads on the reference sequences. Gene quantification analysis and analyses based on gene expression levels (such as principal component analysis, correlation analysis, and differential gene screening) were then performed.

### 2.8. Analysis of DEGs and Functional Enrichment

The obtained RNA-sequence data have been submitted to the NCBI database (PRJNA1423204). Count the number of genes that are up-regulated and down-regulated in each comparison group. Use a Venn diagram to illustrate the differential gene expression among the different comparison groups. To enhance the screening of candidate target genes, prioritize the outcomes of differentially expressed genes following MB treatment. The selection of DEGs was undertaken in accordance with the following criteria: absolute value of expression fold change |log_2_ (FC)| > 1.5 and statistical significance Q < 0.05. To understand the functions of the DEGs, GO functional enrichment and KEGG pathway analysis were carried out by Goatools (https://github.com/tanghaibao/Goatools, accessed on 13 October 2025) and KOBAS (http://www.cbi.pku.edu.cn, accessed on 15 October 2025), respectively. DEGs were significantly enriched in GO terms and metabolic pathways when their Bonferroni-corrected *p*-value was less than 0.05.

### 2.9. Quantitative Real-Time PCR Analysis

Primers were designed using Premier 5.0 software according to the complete open reading frame (ORF) sequence of *AmDld* in the NCBI database. RT-qPCR was then used to detect *AmDld* in different treatment groups. *β-Actin* was chosen as the internal reference gene to determine relative gene expression. The total volume of the reaction was 20 μL, containing 10 μL SYBR Green qPCR Master Mix, 0.4 μL F/R primers, 1 μL cDNA template, and 8.2 μL nuclease-free water. The amplification conditions were as follows: 95 °C for 2 min, 95 °C for 10 s, 60 °C for 30 s, and 40 cycles. The gene expression of each group was calculated by the 2^−ΔΔ T^ method.

### 2.10. Detection of Gene Silencing Efficiency

A segment was selected from the open reading frames of *AmDld*, and primers containing the T7 promoter sequence were designed. The fragments were amplified via PCR, after which the products were recovered and stored at −20 °C. The green fluorescent protein (*GFP*) (GenBank ID: U87974) gene was utilized as a negative control. Double-stranded RNA (dsRNA) was synthesized via a T7 high-efficiency transcription kit (Promega T7 RiboMAX™ Express Large Scale RNA Production System). The negative control comprised 1 μL ds*GFP* (2 μg·μL^−1^), whilst the gene silencing group comprised 1 μL ds*AmDld* (2 μg·μL^−1^). On day 3, 1 μL of dsRNA was injected into the food near the larvae’s heads [19]. Samples were collected on day 4 to assess silencing efficiency across groups. Each experiment was conducted in five biological replicates.

### 2.11. Bioassay of Larvae After Feeding dsRNA

Two groups were set up for experimentation, with each group having four treatment groups and five replicates for each treatment. The larvae in both groups were healthy specimens that were cultured from the 1 to 3-day stage. The experimental treatments commenced once the larvae had reached the 3-day-old stage. TMX was maintained at a concentration of 40 mg·L^−1^, while MB was set at 0.32 mg·L^−1^. One experimental group was fed ds*AmDld*, and the other ds*GFP*, with the ds*GFP*-fed group serving as the control. The administration of dsRNA was carried out on the 3-day-old of age, which was consistent with the treatment time for the silencing efficiency experiment. Larval mortality was recorded on day 5 under standard experimental rearing conditions.

### 2.12. AmDld Enzyme Content Determination

The content of AmDld enzyme was quantified as units per gram (U·g^−1^). Each group comprised five independent biological replicates. Sample preparation was conducted as follows. Initially, the processed sample was subjected to cryopreservation in liquid nitrogen. Then, an appropriate volume of physiological saline solution (0.9% sodium chloride) was added to the frozen samples. A tissue homogenizer was used to grind the mixture of samples and physiological saline thoroughly and uniformly. The prepared crude homogenate was transferred to centrifuge tubes and placed in a high-speed refrigerated centrifuge. Centrifugation was performed at 3000× *g* for 10 min at a temperature of 4 °C, and then the resultant upper layer was meticulously collected and stored at −20 °C.

In the AmDld enzyme content determination phase, a commercial AmDld enzyme-linked immunosorbent assay ELISA Kit (Yuanju Bio) was utilized. The maximum extinction coefficient value of the reaction system was measured at a wavelength of 450 nm using a microplate reader during the detection process. The AmDld enzyme content in the reaction system can be indirectly reflected by detecting the change in absorbance at this wavelength. Subsequently, the specific content of AmDld enzyme in the samples (expressed as U·g^−1^) was calculated by comparing the measured absorbance values with a standard curve (generated using AmDld enzyme standards of known concentrations).

### 2.13. Statistical Analysis

Data analysis was performed using IBM SPSS 20.0 (IBM Corporation, Somers, NY, USA). One-way analysis of variance (ANOVA) with Tukey’s multiple comparisons test and Student’s *t* test were employed. The results are reported as the mean ± SD. Statistical significance was denoted by letters and asterisks: letters indicate significant differences among multiple groups (*p* < 0.05), while the symbols * and ** indicate significance levels of *p* < 0.05 and *p* < 0.01, respectively, for pairwise comparisons. The figures were created using GraphPad Prism 8, Origin Lab 2023b, and the ChiPlot online tool (https://www.chiplot.online/, accessed on 17 October 2025).

## 3. Results

### 3.1. Mitigating Effect of MB on TMX Poisoning in Bees

The larvae were raised according to the standard rearing method for five days (Figure 1A). The mortality rates of each group were then counted. The larval mortality rates were equivalent in the MB treatment group and the control group (Figure 1B, *p* = 0.9990). The mean mortality rate of bee larvae in the TMX-treated group was 47.2% ± 6.4%, while the TMX + MB-C1, -C2, and -C3 groups ranged from 25.0% ± 4.2% to 34.7% ± 6.4% (Figure 1B, *p* < 0.001). Compared with the TMX-treated group, incorporating MB substantially decreased the mean mortality rate of bee larvae. The greatest reduction in average mortality rate of bee larvae was seen in the TMX+MB-C2 group, where the MB concentration was set at 0.32 mg·L^−1^ (*p* = 0.003).

### 3.2. Transcriptome Analysis

All samples yielded high-quality sequencing data. A total of approximately 566.07 million raw reads were generated from the 12 samples. After quality control filtering, 534.25 million valid clean reads were obtained. The proportion of valid sequences ranged from 93.6% to 95.2%. The Q20 and Q30 values were all >98.0% and >93.3%, respectively. The data quality met the requirements for subsequent transcriptome analysis. The reference genome of Italian bees (*Apis_mellifera*_7460.NCBI.GCF_003254395.2_Amel_HAv3.1) was used as the comparison background, and the comparison efficiency of each sample was found to be stable and high. The total comparison rate of all samples ranged from 97.3% to 98.3%, while the unique comparison rate ranged from 94.2% to 96.0%. This indicates that the sequencing data matched the reference genome well and can be used for subsequent gene expression and differential analysis.

### 3.3. DEGs Associated with MB Treatment in Bees

**Figure 2:**
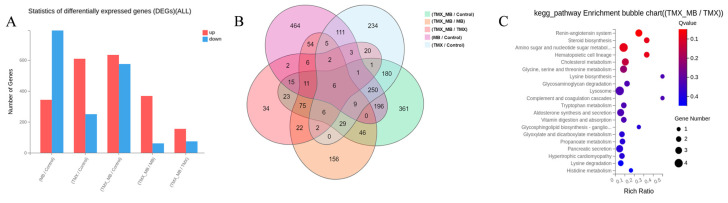
Transcriptome sequencing analysis of larvae under TMX and MB treatment. (**A**) Number of DEGs across groups. (**B**) Overlap of DEGs across groups. (**C**) Pathway enrichment analysis of DEGs between TMX and TMX + MB groups. TMX: thiamethoxam, MB: methylene blue.

Statistical analysis of the number of DEGs in each treatment group revealed that, except for the MB/Control pair, where downregulated genes outnumbered upregulated genes, all other comparisons showed more upregulated genes than downregulated genes. The highest number of DEGs was observed between the TMX + MB/Control groups, while the lowest number was found between the TMX + MB/TMX groups (Figure 2A). Statistical analysis of the DEGs revealed 229 DEGs between the TMX-treated and TMX+MB-treated groups, comprising 155 upregulated DEGs and 74 downregulated DEGs. The results indicate that a significantly greater number of genes exhibited upregulation than downregulation (Figure 2A). The analysis of the Venn diagram revealed 14 overlapping DEGs in the TMX/Control and TMX + MB/Control groups (Figure 2B).

KEGG pathway enrichment analysis revealed the primary biological processes and functions involved in DEGs under TMX + MB and TMX treatment conditions. These pathways cover a wide range of metabolism-related processes, including amino acid, nucleotide sugar, cholesterol, glycine, serine, threonine, lysine, and histidine metabolism, as well as propionate and glycosaminoglycan metabolism. Furthermore, research revealed pathways associated with signalling and cellular processes. These included the renin–angiotensin system, steroid biosynthesis, hematopoietic cell lineages, complement and coagulation cascades, and lysosomal pathways linked to organelle function (Figure 2C). These findings suggest that TMX + MB treatment may exert multifaceted effects on metabolic regulation, signalling, and cellular functions within the organism.

### 3.4. Identification of Potential Genes Associated with MB Treatment

**Figure 3:**
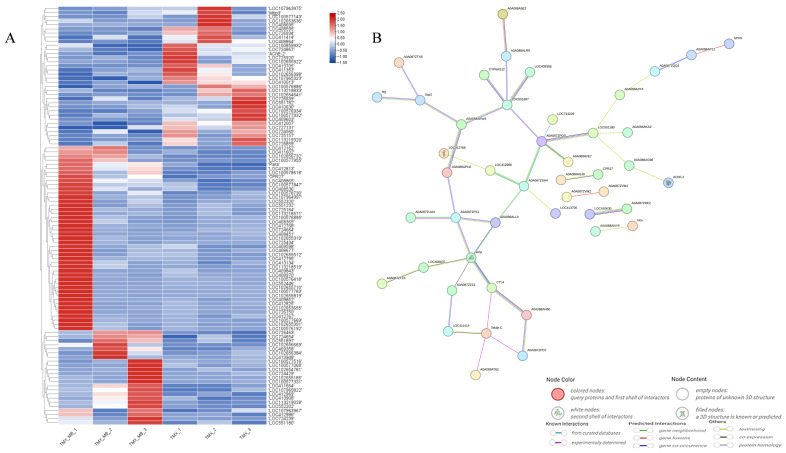
Screening of target genes. (**A**) Gene expression profiles exhibiting absolute fold changes greater than 1.5 between the TMX and TMX + MB groups. (**B**) Predicted protein interaction networks for genes displaying fold changes exceeding 1.5 between the TMX and TMX + MB groups. The threshold for screening differential genes was set at |log_2_ (FC)| ≥ 1.5, Q < 0.05.

The candidate expressed genes in the TMX + MB group and the TMX group were selected based on the condition that the absolute |log_2_ (FC)| > 1.5 and Q < 0.05. A total of 79 genes were screened. These DEGs underwent cluster analysis (Figure 3A) and the prediction of protein interaction networks (Figure 3B). Within the predicted protein interaction networks, AmDld occupied a relatively central position, surrounded by various proteins. This indicates that AmDld occupies a relatively important position among these DEGs. Simultaneously, searching the KEGG database for pathways involving *AmDld* gene revealed that 5 of the 15 reported pathways matched the KEGG analysis results of the TMX + MB and TMX groups’ transcriptomes.

### 3.5. Spatial Expression Patterns of AmDld in Larval Tissues

**Figure 4:**
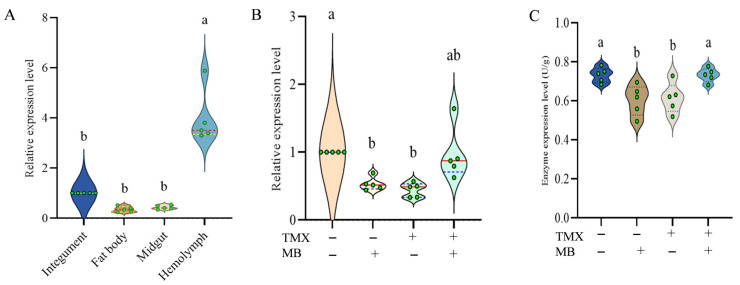
Comparisons of the expression levels and enzyme content of *AmDld*. (**A**) Expressions of *AmDld* among four tissues of 5-day-old larvae (*n* = 5, *p* < 0.001). (**B**) Expressions of *AmDld* across different treatment groups (*n* = 5, *p* = 0.008). (**C**) Enzyme content of AmDld in different treatment groups (*n* = 5, *p* = 0.0034). *AmDld*: *Apis mellifera* dihydrolipoyl dehydrogenase.

The expression levels of *AmDld* were compared among four different tissues. As the results, the expression of *AmDld* was lower in the fat body, midgut, and integument (*p* > 0.05). However, the expression of *AmDld* in the hemolymph was over three times higher than in the integument (Figure 4A, *p* < 0.0001).

### 3.6. Alteration of AmDld Gene Expression and Its Enzyme Content Under TMX and MB Treatment

Compared with the control group, *AmDld* expression levels were significantly reduced in both the MB (*p* = 0.0131) and TMX (*p* = 0.0035) groups. The expression level of *AmDld* was comparable between the MB + TMX (*p* > 0.05) group and the control group (Figure 4B).

Enzyme content of AmDld was measured in each group following standard experimental treatment using an ELISA kit (Shanghai Enlink Biotechnology Co., Ltd., Shanghai, China). Results indicated that enzyme content in the TMX (*p* = 0.0337) and MB (*p* = 0.0184) groups exhibited significant inhibition compared to the control. Furthermore, no discernible difference was observed in enzyme content between the control and TMX + MB groups (Figure 4C, *p* = 0.9949).

### 3.7. Silence of AmDld and the Effect on Its Enzyme Content

**Figure 5:**
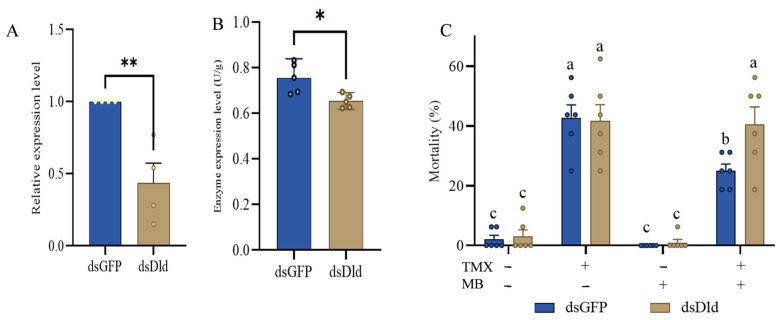
Knockdown and function of *AmDld* in bee larvae. (**A**) Silencing efficiency following dsRNA feeding (*n* = 4, *p* < 0.0001; 95% CI: −0.9027 to −0.2273). (**B**) Differences in enzyme content after *AmDld* knockdown (*n* = 5, *p* = 0.0143; 95% CI: −0.1785 to −0.02668). (**C**) Changes in larval mortality rates following dsRNA feeding under different treatments (*n* = 5, *p* = 0.0360). Data were analyzed using the one-way analysis of variance (ANOVA), with Tukey’s multiple comparisons test, and Student’s *t* test were employed. Bars represent mean ± SD, and asterisks * indicate *p* < 0.05, ** indicate *p* < 0.01. Differences between groups are denoted by letters, with *p* < 0.05 representing the threshold for statistical significance.

The relative expression levels of *AmDld* in the two groups of larvae were detected and quantitatively analyzed using the quantitative real-time polymerase chain reaction (qRT-PCR) technique. The results showed that the content of *AmDld* was significantly lower in the larvae treated with ds*AmDld* than in the control group (Figure 5A, *p* < 0.0001). Specifically, the relative expression levels of *AmDld* in the treatment group was 56.5% lower than those in the control group.

Moreover, the enzymatic content of AmDld in larvae following ds*AmDld* interference was assessed. The results indicated that the enzymatic content of AmDld was effectively suppressed after knockdown of *AmDld* (Figure 5B, *p* = 0.0143).

### 3.8. The Mitigation of TMX Toxicity by MB Is Mediated by AmDld

Following administration of dsRNA, no significant difference in mortality was observed between the control and MB groups, with mortality rates in both groups remaining below 10%. Following the administration of ds*AmDld*-containing feed, the mortality of larvae in the TMX + MB group increased to 40.6%, whereas the ds*GFP* group exhibited a mortality rate of 25.0%, which was significantly lower than that in TMX + MB group, (Figure 5C, *p* = 0.0360).

## 4. Discussion

The larval stage of bees is crucial for the continuation and development of the colony. The normal development of the larvae directly affects the rate at which the colony is replenished, its size, and its production capacity. Only when the larvae develop normally can the colony continuously produce new bees and maintain a division of labour for activities such as foraging, feeding and nest protection. This ensures the colony’s stability and reproductive potential. Therefore, the health of the larvae is an important factor in assessing the impact of chemical substances and environmental factors on bees. The aim of using a feeding dose of 40 mg·L^−1^ for bee larvae was to simulate the intake of 2000 ng of TMX per bee after the larvae were three or four days old. The survival probability of the larvae on the fifth day was approximately 50% [20]. The protective effect of MB can be better verified and highlighted by conducting experiments with a higher intake of TMX. Furthermore, studies have shown that larvae are substantially more resistant to TMX than worker bees [21]. Systemic insecticides rank among the most hazardous chemicals, such as TMX, thiacloprid, and imidacloprid. These are more likely than any other insecticides to cause toxic effects in worker bees and larvae [22,23]. However, there are currently no definitive reports providing effective methods to alleviate bee poisoning from TMX.

KEGG analysis annotation results show that metabolic pathways, signalling pathways and cell pathways, as well as the steroid biosynthesis pathway, play significant roles in protecting bee larvae from TMX poisoning. Amino acid metabolism and glycine, serine, and threonine metabolism are highly correlated with the synthesis of antioxidant substances and energy metabolism. These pathways can work in synergy with AmDld (a key enzyme in mitochondrial energy metabolism) to restore the energy supply of larvae under TMX stress. The complement and coagulation cascade reactions and the lysosomal pathway are involved in immune responses and the degradation of toxic substances, reducing cell damage caused by TMX. The steroid biosynthesis pathway regulates insect tissue development and alleviates larval developmental inhibition caused by TMX.

MB is a phenothiazine with a low redox potential that can cycle between oxidized (MB+) and reduced (leuco MB) forms [24]. This process enables electrons to be transferred directly from NADH/NADPH to cytochrome c or oxygen molecules, thereby bypassing complex I [25]. Consequently, MB demonstrates considerable promise in safeguarding bees against the consequences of pesticide poisoning and in the restoration of energy metabolism. This hypothesis was further supported by our experimental findings. In the experiment, the mortality rate of TMX-fed larvae was observed to decrease from 47.2% to 25.0% following the administration of MB supplementation. This result provides additional evidence confirming the inference that MB serves as a key substance in alleviating pesticide poisoning.

Although MB has been demonstrated to be effective in the treatment of cyanide poisoning and acquired methemoglobinemia (MetHB), the precise mechanisms by which it exerts its detoxifying effects remain to be elucidated [9,10]. The findings of the research suggest that MB has the potential to act as an antidote for the mitochondrial toxicity induced by pesticides in hornets. It was found that MB stimulates mitochondrial respiration mediated by electron transport chain (ETC) complex I, restoring the mitochondrial membrane potential dissipated by pesticide-induced ETC disruption [12]. Consequently, material energy metabolism may be key to MB’s detoxification function. The *AmDld* gene primarily encodes Dihydrolipoamide dehydrogenase, an enzyme that functions as a mitochondrial protein in at least five multienzyme complexes, playing a role in eukaryotic energy metabolism. Consequently, the detoxifying action of MB is inextricably linked to the *AmDld* gene.

The concomitant administration of MB and TMX has been demonstrated to restore *AmDld* enzyme content to near-normal levels, suggesting that MB intervenes via a direct or indirect mechanism. The hypothesis that MB, as a distinct redox cycler, alleviates TMX-induced oxidative stress, thereby stabilizing or protecting the AmDld protein, is postulated. It is hypothesized that MB may play a role in maintaining cellular redox homeostasis. This, in turn, would safeguard *AmDld* catalytic function and ensure metabolic steadiness when confronted with toxic challenges.

When the expression level of *AmDld* returns to the level of the control group, the mortality rate of the larvae significantly decreases, and the key genes such as *CYP450, AChE*, and *FPPS*, which are differentially expressed, are also closely related. Cytochrome P450 (*CYP450*) is a superfamily of enzymes that plays a crucial role in the metabolism of drugs, steroids, fat-soluble vitamins, carcinogens, pesticides, and many other chemicals [26]. Acetylcholinesterase is involved in the development and maturation of cells and can promote the development of neurons and nerve regeneration [27]. Farnesyl pyrophosphate synthase (*FPPS*) is a key enzyme in the mevalonate pathway, which is the exclusive route of isoprenoid production in animals. It is involved in the biosynthesis of cholesterol and intermediates that are important for intracellular signalling and growth control [28]. These genes play a significant role in the metabolism of detoxification, the protection and regeneration of the nervous system, the synthesis and supply of proteins and energy, and the construction of cell membranes. In the future, we plan to use weighted gene co-expression network analysis (WGCNA) to identify additional candidate genes and verify the combined impact of various genes and pathways involved in the detoxification process. Hemolymph is the circulatory fluid within the hemocoel of invertebrates such as arthropods and molluscs. It is composed of plasma and blood cells. Its functions include transporting nutrients and metabolic waste products, maintaining osmotic pressure equilibrium, and participating in immune defence [29]. The elevated expression of *AmDld* in hemolymph serves to further validate the gene’s pivotal role in energy metabolism and material transformation.

The protective effect of *AmDld* was determined by functional validation through RNAi, which provided substantial evidence. The experiments used non-invasive methods instead of RNAi for the purpose [19]. The feeding volume of the larvae at the 3rd and 4th days of age is approximately 20–30 μL [30]. We believe that the larvae can completely consume the 1 μL dsRNA added near their heads to ensure the silencing efficiency. The knockdown of *AmDld* expression rendered the larvae more susceptible to TMX toxicity, thereby confirming that the integrity of gene function is a determining factor of survival.

It seems that the main way TMX works is by stopping the action of nicotinic acetylcholinesterase receptors in the insect nervous system. This results in the disruption of normal neural signalling, ultimately leading to the death of the pest due to paralysis [31]. In insects experiencing paralysis, there is a persistent muscle twitching phenomenon. This is intrinsically linked to mitochondrial energy supply and metabolic processes. MB has been demonstrated to maintain normal electron transfer and energy metabolism through an unconventional electron transport chain [25]. This effectively compensates for the loss of the *AmDld* gene function. The sustained energy supply in question has been demonstrated to permit insects to undergo additional metabolic transformation of the pesticide, thereby achieving effective mitigation of its toxicity. Although the current research lacks direct interactions and validations at the mitochondrial functional level and has not involved direct mechanistic experiments such as co-immunoprecipitation, protein stability, mitochondrial membrane potential, oxygen consumption rate and reactive oxygen species levels, nevertheless, the synergy between RNAi and enzyme content data strongly supports the model that TMX partially damages mitochondrial energy metabolism by inhibiting *AmDld*. Verifying mitochondrial membrane potential, respiratory function, reactive oxygen species levels and protein interactions will be a key area of research in the future.

During the breeding and protection of bees, MB should be used rationally, based on the principle of prevention. When bee poisoning incidents or pesticide use in fields occur frequently, a suitable amount of MB can be added to sugar water or pollen provided as rewards or supplementary feed for bees, to prevent and protect them. Currently, the laboratory is testing the protective effect of MB on bee colonies (worker bees) in the field. This involves detecting changes in foraging behaviour, survival rates and mitochondrial function in adult worker bees after combined use with TMX, as well as exploring application methods of MB that are suitable for protecting adult worker bees. We expect to achieve excellent results. In the future, we hope to clarify the relationship between MB intake and larval intake, analyze the applicable MB concentration, the frequency of use in actual field conditions, and its potential impact on bee colonies. We aim to provide scientific guidance on the use of MB to protect bees from TMX poisoning.

## 5. Conclusions

The results demonstrate the establishment of a novel detoxification pathway, whereby MB reduces the toxicity of TMX to bee larvae by targeting and restoring the function of *AmDld*. This study has not only deepened our understanding of the molecular mechanism of neonicotinoid insecticide poisoning but also highlighted the potential of MB as a therapeutic agent or protective supplement for the bee industry. It is recommended that future studies concentrate on elucidating the precise molecular interactions between MB and AmDld proteins, and that these findings be validated in field-level studies of adult bees.

## Data Availability

The original contributions presented in this study are included in the article/Supplementary Materials. Further inquiries can be directed to the corresponding authors.

## Supplementary Materials

The following supporting information can be downloaded at: https://www.mdpi.com/article/10.3390/insects17030334/s1, Figure S1: Principal component analysis of all samples (Apis mellifera larvae); Table S1: Reads filtering and quality control statistics for RNA-seq libraries; Table S2: Statistics of read mapping to the reference genome; Table S3: Differential gene information.

## References

[1] Fernandes K.E., Stanfield B., Frost E.A., Shanahan E.R., Susantio D., Dong A.Z., Tran T.D., Cokcetin N.N., Carter D.A. (2023). Low levels of hive stress are associated with decreased honey activity and changes to the gut microbiome of resident honey bees. Microbiol. Spectr..

[2] Breda D., Frizzera D., Giordano G., Seffin E., Zanni V., Annoscia D., Topping C.J., Blanchini F., Nazzi F. (2022). A deeper understanding of system interactions can explain contradictory field results on pesticide impact on honey bees. Nat. Commun..

[3] Furlan L., Kreutzweiser D. (2015). Alternatives to neonicotinoid insecticides for pest control: Case studies in agriculture and forestry. Environ. Sci. Pollut. Res..

[4] Dively G.P., Kamel A. (2012). Insecticide residues in pollen and nectar of a cucurbit crop and their potential exposure to pollinators. J. Agric. Food Chem..

[5] Easton A.H., Goulson D. (2013). The neonicotinoid insecticide imidacloprid repels pollinating flies and beetles at field-realistic concentrations. PLoS ONE.

[6] Tasman K., Hidalgo S., Zhu B., Rands S.A., Hodge J.J.L. (2021). Neonicotinoids disrupt memory, circadian behaviour and sleep. Sci. Rep..

[7] Henry M., Béguin M., Requier F., Rollin O., Odoux J.F., Aupinel P., Aptel J., Tchamitchian S., Decourtye A. (2012). A common pesticide decreases foraging success and survival in honey bees. Science.

[8] Desneux N., Sandrock C., Tanadini M., Tanadini L.G., Fauser-Misslin A., Potts S.G., Neumann P. (2014). Impact of chronic neonicotinoid exposure on honeybee colony performance and queen supersedure. PLoS ONE.

[9] Pushparajah Mak R.S., Liebelt E.L. (2021). Methylene blue an antidote for methemoglobinemia and beyond. Pediatr. Emerg. Care.

[10] Haouzi P., McCann M., Tubbs N., Judenherc-Haouzi A., Cheung J., Bouillaud F. (2019). Antidotal effects of the phenothiazine chromophore methylene blue following cyanide intoxication. Toxicol. Sci..

[11] Delport A., Harvey B.H., Petzer A., Petzer J.P. (2018). Methylene blue analogues with marginal monoamine oxidase inhibition retain antidepressant-like activity. ACS Chem. Neurosci..

[12] Syromyatnikov M., Nesterova E., Smirnova T., Popov V. (2021). Methylene blue can act as an antidote to pesticide poisoning of bumble bee mitochondria. Sci. Rep..

[13] Bouillaud F., Ransy C., Moreau M., Benhaim J., Lombès A., Haouzi P. (2022). Methylene blue induced o2 consumption is not dependent on mitochondrial oxidative phosphorylation: Implications for salvage pathways during acute mitochondrial poisoning. Respir. Physiol. Neurobiol..

[14] Zhou L., Flores J., Noël A., Beauchet O., Sjöström P.J., LeBlanc A.C. (2019). Methylene blue inhibits caspase-6 activity, and reverses caspase-6-induced cognitive impairment and neuroinflammation in aged mice. Acta Neuropathol. Commun..

[15] Ou G., Che J., Dong J., Deng Y., Jiang X., Sun Y., He Z., Chen W., Zhang J. (2023). Methylene blue targets phd3 expression in murine microglia to mitigate lipopolysaccharide-induced neuroinflammation and neurocognitive impairments. Int. Immunopharmacol..

[16] Aynalem T., Meng L., Getachew A., Wu J., Yu H., Tan J., Li N., Xu S. (2024). A new isolated fungus and its pathogenicity for *Apis mellifera* brood in china. Microorganisms.

[17] Gong Z.-X., Cheng F.-P., Xu J.-N., Yan W.-Y., Wang Z.-L. (2023). The juvenile-hormone-responsive factor amkr-h1 regulates caste differentiation in honey bees. Biomolecules.

[18] Gao Q., Wu Y.F., Huang J.N., Xu S.W., Chen R.H., Jiang H.B., Huang Y., He Q.B., Wang Y.H., Xiao J.J. (2026). Chronic neonicotinoid exposure disrupts survival, development, digestive enzymes, and gut microbiome in honeybee queen larvae (*Apis mellifera* L.). Pestic. Biochem. Physiol..

[19] Nunes F.M.F., Simoes Z.L.P. (2009). A non-invasive method for silencing gene transcription in honeybees maintained under natural conditions. Insect Biochem. Mol. Biol..

[20] Grillone G., Laurino D., Manino A., Porporato M. (2017). Toxicity of thiametoxam on in vitro reared honey bee brood. Apidologie.

[21] Oliveira R.A., Roat T.C., Carvalho S.M., Malaspina O. (2013). Side-effects of thiamethoxam on the brain andmidgut of the africanized honeybee *Apis mellifera* (Hymenopptera: Apidae). Environ. Toxicol..

[22] Chaline N., Yang E.-C., Chang H.-C., Wu W.-Y., Chen Y.-W. (2012). Impaired olfactory associative behavior of honeybee workers due to contamination of imidacloprid in the larval stage. PLoS ONE.

[23] Guedes R.N.C., Sanchez-Bayo F., Goka K. (2014). Pesticide Residues and Bees–A Risk Assessment. PLoS ONE.

[24] Atamna H., Mackey J., Dhahbi J.M. (2012). Mitochondrial pharmacology: Electron transport chain bypass as strategies to treat mitochondrial dysfunction. BioFactors.

[25] Xue H., Thaivalappil A., Cao K. (2021). The Potentials of Methylene Blue as an Anti-Aging Drug. Cells.

[26] Denison M.S., Whitlock J.P. (1995). Xenobiotic-inducible Transcription of Cytochrome P450 Genes. J. Biol. Chem..

[27] Li F., Guo S., Zhang S., Peng S., Cao W., Ho C.-T., Bai N. (2019). Bioactive Constituents of *F. esculentum* Bee Pollen and Quantitative Analysis of Samples Collected from Seven Areas by HPLC. Molecules.

[28] Buhaescu I., Izzedine H. (2007). Mevalonate pathway: A review of clinical and therapeutical implications. Clin. Biochem..

[29] Blow F., Douglas A.E. (2019). The hemolymph microbiome of insects. J. Insect Physiol..

[30] Slater G.P., Yocum G.D., Bowsher J.H. (2020). Diet quantity influences caste determination in honeybees (*Apis mellifera*). Proc. R. Soc. B-Biol. Sci..

[31] Zhang A.N., Zhou W.J., Wu D.X., Han L.L., Zhao K.J. (2022). Effects of multigenerational imidacloprid and thiamethoxam stress on metabolism and physiology of *Aphis glycines* Matsumura (Hemiptera: Aphididae). PLoS ONE.

