# Supplementary material for: Methylene Blue Alleviates Thiamethoxam-Induced Toxicity in Honeybee Larvae by Activating Dihydrolipoyl Dehydrogenase
Source: Insects. 2026 Mar 19;17(3):334. doi: 10.3390/insects17030334 (PMC13026182; doi:10.3390/insects17030334)
Supplement: Supplementary file 1 [file insects-17-00334-s001.zip › insects-4171463-supplementary.pdf]

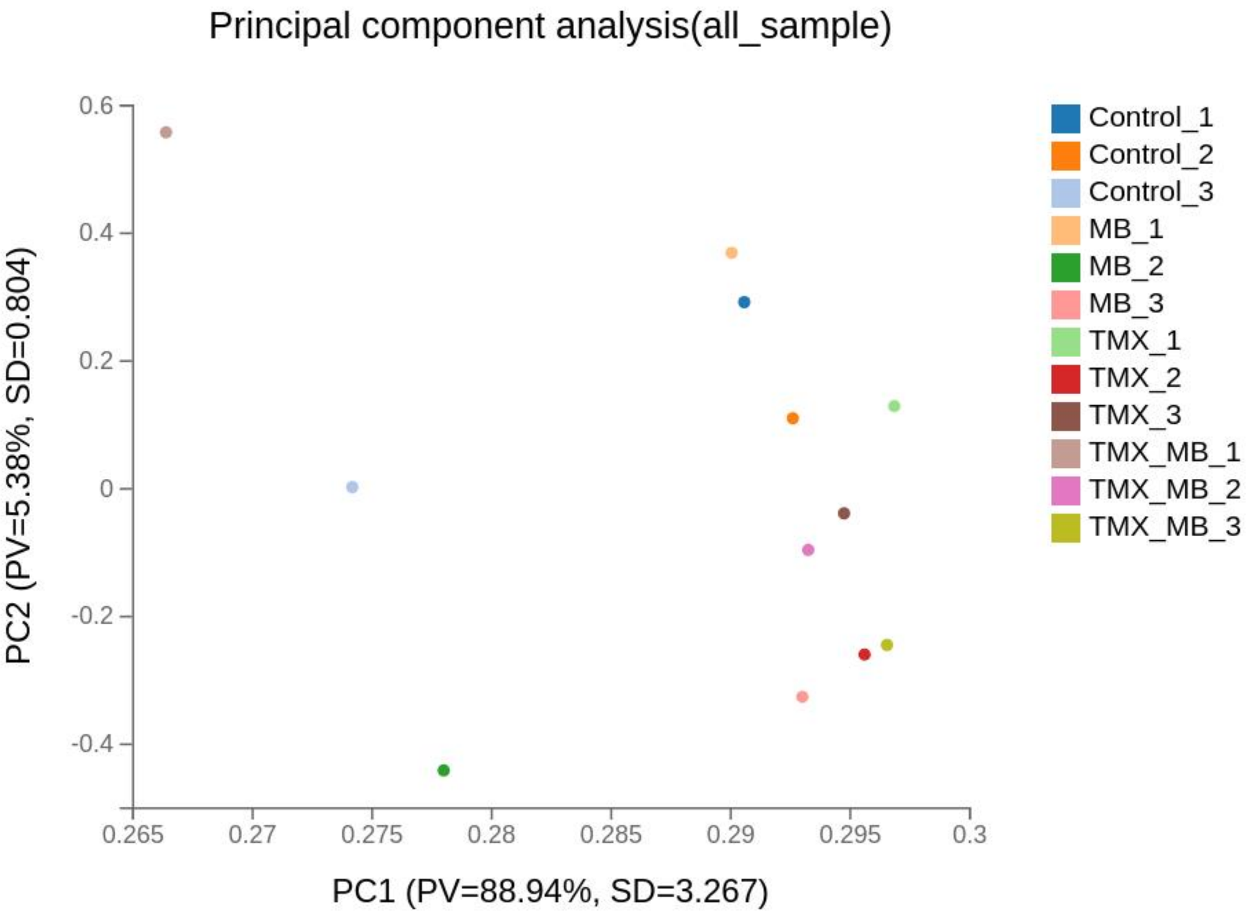

Figure S1: Principal component analysis of all samples (Apis mellifera larvae)

Table S1: Reads filtering and quality control statistics for RNA-seq libraries

sample	total_raw_reads (M)	total_clean_reads (M)	total_clean_bases (Gb)	clean_reads_q20 (%)	clean_reads_q30 (%)	clean_reads_ratio (%)
Control_1	47.19	44.9	6.73	98.19	93.81	95.15
Control_2	46.98	44.6	6.69	98.15	93.62	94.92
Control_3	47.19	44.2	6.63	98.06	93.38	93.66
MB_1	47.19	44.43	6.66	98.16	93.73	94.17
MB_2	47.19	44.44	6.67	98.12	93.62	94.19
MB_3	47.19	44.55	6.68	98.03	93.32	94.41
TMX_1	47.19	44.49	6.67	98.44	94.5	94.29
TMX_2	47.19	44.15	6.62	98.41	94.42	93.58
TMX_3	47.19	44.76	6.71	98.33	94.11	94.86
TMX_MB_1	47.19	44.8	6.72	98.31	94.08	94.95
TMX_MB_2	47.19	44.73	6.71	98.38	94.3	94.8
TMX_MB_3	47.19	44.2	6.63	98.47	94.61	93.67

Table S2: Statistics of read mapping to the reference genome

(Reference genome version:Apis_mellifera_7460.NCBI.GCF_003254395.2_Amel_HAv3.1.v2201)

sample	total_clean_read(M)	total_mapping_genome_ratio(%)	uniquely_mapping_genome_ratio(%)
Control_1	44.9	97.97	95.42
Control_2	44.6	98.31	95.97
Control_3	44.2	97.53	94.22
MB_1	44.43	97.25	94.43
MB_2	44.44	97.76	94.6
MB_3	44.55	97.59	94.73
TMX_1	44.49	97.72	94.9
TMX_2	44.15	97.37	94.55
TMX_3	44.76	98.09	95.18
TMX_MB_1	44.8	98.22	94.93
TMX_MB_2	44.73	98.01	94.84
TMX_MB_3	44.2	97.79	95.31

Table S3: Differential gene information

Gene ID	Gene Symbol	Type	log2 (TMX_MB / TMX)	Qvalue (TMX_MB / TMX)	GenBank Desc
102655185	'LOC102655185'	mRNA	8.803512187	1.30E-43	serine-rich adhesin for platelets-like
412162	'LOC412162'	mRNA	5.177104968	6.26E-43	armadillo repeat-containing protein gudu
411664	'LOC411664'	mRNA	4.153258226	1.21E-04	uncharacterized LOC411664
100578618	'LOC100578618'	mRNA	4.070796066	2.15E-04	uncharacterized LOC100578618
100577331	'LOC100577331'	mRNA	3.943193834	7.22E-263	cell wall integrity and stress response component 1
102656669	'LOC102656669'	mRNA	3.687877341	5.12E-208	ctenidin-1-like
102656732	'LOC102656732'	mRNA	3.386457402	1.27E-37	uncharacterized LOC102656732
102653655	'LOC102653655'	mRNA	3.181602852	2.00E-45	mucin-19
113219028	'LOC113219028'	mRNA	2.975340434	2.86E-236	pancreatic triacylglycerol lipase-like
551232	'LOC551232'	mRNA	2.790688147	3.54E-06	probable nuclear hormone receptor HR38
102655391	'LOC102655391'	mRNA	2.75732955	1.27E-26	dihydrolipoyl dehydrogenase, mitochondrial
725164	'LOC725164'	mRNA	2.683772943	1.51E-06	serine-rich adhesin for platelets
102656384	'LOC102656384'	mRNA	2.61560144	2.93E-05	dual specificity protein phosphatase 19
100577955	'LOC100577955'	mRNA	2.592142467	4.95E-18	odorant receptor 13a
409018	'Para'	mRNA	2.38884795	1.06E-39	sodium channel protein paralytic
552202	'LOC552202'	mRNA	2.380223377	4.66E-21	monocarboxylate transporter 9
413948	'LOC413948'	mRNA	2.375650647	3.16E-14	tektin-1
408451	'LOC408451'	mRNA	2.310062306	4.63E-38	NADPH oxidase 5
411602	'LOC411602'	mRNA	2.30526132	6.64E-33	dynein heavy chain 6, axonemal
724429	'LOC724429'	mRNA	2.227640422	2.30E-24	synaptic vesicle glycoprotein 2C-like

100576192	'LOC100576192'	mRNA	2.076442629	9.24E-08	uncharacterized LOC100576192
102655319	'LOC102655319'	mRNA	2.023348904	2.59E-35	inner centromere protein A
409598	'LOC409598'	mRNA	2.015754702	1.58E-05	uncharacterized LOC409598
726463	'LOC726463'	mRNA	1.94269124	2.52E-04	inositol monophosphatase 2
100577669	'LOC100577669'	mRNA	1.934423624	5.86E-87	uncharacterized LOC100577669
102655512	'LOC102655512'	mRNA	1.89022382	1.97E-09	uncharacterized LOC102655512
107965822	'LOC107965822'	mRNA	1.889985141	5.71E-146	retrovirus-related Pol polyprotein from type-1 retrotransposable element R2
412813	'LOC412813'	mRNA	1.852891036	2.73E-37	zwei Ig domain protein zig-8
412986	'LOC412986'	mRNA	1.814733621	9.56E-23	putative fatty acyl-CoA reductase
552320	'LOC552320'	mRNA	1.787782416	7.84E-23	uncharacterized LOC552320
100578730	'LOC100578730'	mRNA	1.784787545	1.29E-11	uncharacterized LOC100578730
413134	'LOC413134'	mRNA	1.735405711	4.47E-28	UNC93-like protein
100577847	'LOC100577847'	mRNA	1.728760398	1.82E-10	uncharacterized LOC100577847
724654	'LOC724654'	mRNA	1.721975397	3.18E-49	cytochrome b5
412825	'LOC412825'	mRNA	1.681172488	5.34E-80	sushi, von Willebrand factor type A, EGF and pentraxin domain-containing protein 1
100577516	'LOC100577516'	mRNA	1.679804041	2.33E-30	venom acid phosphatase Acph-1
100577068	'LOC100577068'	mRNA	1.677230097	2.65E-05	odorant receptor 4-like
100576895	'LOC100576895'	mRNA	1.662339215	6.66E-150	putative fatty acyl-CoA reductase CG5065
412795	'LOC412795'	mRNA	1.651953654	1.22E-51	cadherin-99C
413908	'LOC413908'	mRNA	1.643846758	1.50E-08	cytochrome P450 6A1
113218519	'LOC113218519'	mRNA	1.642296307	5.48E-15	uncharacterized LOC113218519
408865	'LOC408865'	mRNA	1.640445511	1.28E-26	uncharacterized LOC408865
725509	'CPR27'	mRNA	1.614688631	2.76E-37	cuticular protein 27
725434	'LOC725434'	mRNA	1.579283555	3.24E-99	protein artichoke

408536	'LOC408536'	mRNA	1.568295725	5.99E-04	angiotensin-converting enzyme
113218571	'LOC113218571'	mRNA	1.568295725	4.06E-08	hydrocephalus-inducing protein-like
107963967	'LOC107963967'	mRNA	1.554081866	3.64E-08	serine protease inhibitor 3
409358	'LOC409358'	mRNA	1.53082102	3.09E-06	intraflagellar transport protein 74 homolog
551897	'LOC551897'	mRNA	1.525591274	2.68E-17	Bardet-Biedl syndrome 4 protein homolog
412767	'LOC412767'	mRNA	1.509402036	7.55E-06	protein takeout
409759	'LOC409759'	mRNA	1.495832528	5.68E-179	serine-rich adhesin for platelets
100578649	'LOC100578649'	mRNA	1.489134242	8.38E-221	uncharacterized LOC100578649
551920	'LOC551920'	mRNA	1.453917704	8.02E-61	mucin-5AC
100576182	'LOC100576182'	mRNA	1.451442284	1.78E-80	pupal cuticle protein G1A
102654795	'LOC102654795'	mRNA	1.436790942	2.08E-16	putative uncharacterized protein DDB_G0281733
100578363	'LOC100578363'	mRNA	1.435845429	3.25E-04	protein Ycf2
102653881	'LOC102653881'	mRNA	1.405566225	3.31E-09	glucose dehydrogenase [FAD, quinone]-like
726690	'LOC726690'	mRNA	1.390242221	8.60E-162	probable cytochrome P450 6a14
409228	'LOC409228'	mRNA	1.375727063	2.23E-144	scavenger receptor class B member 1
726798	'LOC726798'	mRNA	1.374911751	1.38E-15	sn1-specific diacylglycerol lipase beta
413481	'LOC413481'	mRNA	1.365780927	3.94E-142	probable chitinase 10
410009	'LOC410009'	mRNA	1.36568862	7.34E-28	cadherin-related tumor suppressor
413113	'LOC413113'	mRNA	1.348029605	6.57E-18	probable serine/threonine-protein kinase samkC
726288	'LOC726288'	mRNA	1.346737956	2.64E-15	uncharacterized LOC726288
410976	'LOC410976'	mRNA	1.345903304	3.31E-08	nose resistant to fluoxetine protein 6
726995	'LOC726995'	mRNA	1.343735467	4.81E-23	endocuticle structural glycoprotein ABD-4
413345	'LOC413345'	mRNA	1.330352301	3.65E-09	suppressor APC domain-containing protein 2
726803	'LOC726803'	mRNA	1.311955972	6.43E-07	uncharacterized LOC726803
552276	'LOC552276'	mRNA	1.303071502	9.24E-47	uncharacterized LOC552276
552724	'LOC552724'	mRNA	1.302397611	6.90E-198	zinc finger CCCH domain-containing protein 13
100578248	'LOC100578248'	mRNA	1.29580099	7.84E-22	uncharacterized serine-rich protein C215.13
725239	'LOC725239'	mRNA	1.291840804	2.81E-30	RNA-binding protein 33

551224	'LOC551224'	mRNA	1.279985993	1.91E-48	aminopeptidase N
551280	'LOC551280'	mRNA	1.2643203	8.79E-26	uncharacterized LOC551280
100578913	'LOC100578913'	mRNA	1.254122471	2.61E-26	uncharacterized LOC100578913
410334	'LOC410334'	mRNA	1.2527939	6.59E-08	uncharacterized LOC410334
725547	'LOC725547'	mRNA	1.243047272	5.07E-91	extensin
409821	'LOC409821'	mRNA	1.240227434	1.19E-90	twitchin
725590	'LOC725590'	mRNA	1.239672978	2.82E-05	homeobox protein B-H2-like
413740	'LOC413740'	mRNA	1.234141328	1.91E-10	iodotyrosine deiodinase 1
413168	'LOC413168'	mRNA	1.227969918	2.74E-224	retinol dehydrogenase 14
725305	'LOC725305'	mRNA	1.212579317	2.48E-11	uncharacterized LOC725305
550671	'LOC550671'	mRNA	1.195801376	2.90E-147	venom serine protease Bi-VSP
410756	'LOC410756'	mRNA	1.194585088	1.85E-15	regulating synaptic membrane exocytosis protein 1
725106	'LOC725106'	mRNA	1.193368439	7.12E-16	UDP-glucuronosyltransferase 1-8
112939925	'LOC112939925'	mRNA	1.191958765	3.42E-19	cytochrome P450 6a13-like
725415	'LOC725415'	mRNA	1.19144142	2.53E-07	BTB/POZ domain-containing protein KCTD16
552459	'LOC552459'	mRNA	1.178053228	8.46E-191	glycine-rich cell wall structural protein
102656439	'LOC102656439'	mRNA	1.170351316	6.31E-63	adenosine deaminase 2
727197	'CPR2'	mRNA	1.169682357	1.89E-184	cuticular protein 2
410021	'LOC410021'	mRNA	1.167757796	2.27E-04	protein Skeletor, isoforms B/C
100579026	'LOC100579026'	mRNA	1.166197282	8.88E-05	uncharacterized LOC100579026
406146	'LOC406146'	mRNA	1.159426235	1.20E-41	hyaluronoglucosaminidase
102655706	'LOC102655706'	mRNA	1.159410453	1.02E-11	uncharacterized LOC102655706
724477	'LOC724477'	mRNA	1.158775231	5.89E-13	vitamin K-dependent protein C
408264	'LOC408264'	mRNA	1.156521352	1.18E-149	netrin receptor UNC5C
102655220	'LOC102655220'	mRNA	1.147720043	1.28E-18	uncharacterized LOC102655220
408807	'LOC408807'	mRNA	1.141533267	4.61E-16	uncharacterized LOC408807
100578776	'LOC100578776'	mRNA	1.135336318	2.52E-05	uncharacterized LOC100578776
408830	'LOC408830'	mRNA	1.13369596	4.65E-27	obscurin

102656882	'LOC102656882'	mRNA	1.12853114	1.61E-17	cytochrome P450 9e2-like
410065	'Cpap3-d'	mRNA	1.121245695	9.96E-188	cuticular protein analogous to peritrophins 3-D
552277	'LOC552277'	mRNA	1.117357275	4.59E-168	class E basic helix-loop-helix protein 22
409786	'LOC409786'	mRNA	1.116988277	5.22E-27	jazigo
100576504	'LOC100576504'	mRNA	1.115436761	8.36E-08	uncharacterized LOC100576504
409624	'LOC409624'	mRNA	1.112249253	7.83E-33	acetyl-coenzyme A synthetase
100578072	'LOC100578072'	mRNA	1.089784166	6.80E-21	uncharacterized LOC100578072
413543	'LOC413543'	mRNA	1.087669884	5.19E-17	sushi, von Willebrand factor type A, EGF and pentraxin domain-containing protein 1
409999	'LOC409999'	mRNA	1.086809345	2.49E-18	lipase 3
725178	'LOC725178'	mRNA	1.08504286	4.72E-96	chitooligosaccharidolytic beta-N-acetylglucosaminidase
408596	'LOC408596'	mRNA	1.074076724	5.93E-71	clavesin-2
107965756	'LOC107965756'	mRNA	1.065795385	9.01E-04	uncharacterized LOC107965756
409778	'LOC409778'	mRNA	1.065335738	3.29E-38	storkhead-box protein 2
725041	'LOC725041'	mRNA	1.059282078	5.55E-04	leucine-rich repeat-containing protein 15
410293	'LOC410293'	mRNA	1.051670354	6.05E-12	collectin-11
551490	'LOC551490'	mRNA	1.047463562	2.29E-05	collagen alpha chain CG42342
113218574	'LOC113218574'	mRNA	1.046250521	1.35E-286	mucin-2-like
410870	'LOC410870'	mRNA	1.039916753	2.37E-43	proton channel OtopLc
726252	'LOC726252'	mRNA	1.039186459	3.68E-16	titin
410902	'18-w'	mRNA	1.035172156	2.15E-07	18-wheeler
406114	'LOC406114'	mRNA	1.033959298	2.61E-05	alpha-amylase
410368	'LOC410368'	mRNA	1.033731077	1.22E-35	cadherin-23
724865	'LOC724865'	mRNA	1.032132523	1.63E-35	ABC transporter G family member 20
726418	'LOC726418'	mRNA	1.016437704	6.73E-102	flavin-containing monooxygenase FMO GS-OX-like 4
725804	'LOC725804'	mRNA	1.016070011	3.40E-24	cuticle protein 18.7
410517	'LOC410517'	mRNA	1.005946463	7.51E-96	extracellular serine/threonine protein CG31145
552836	'LOC552836'	mRNA	1.00448306	1.73E-18	uncharacterized LOC552836
100578770	'LOC100578770'	mRNA	1.000581706	2.48E-22	uncharacterized LOC100578770
412646	'LOC412646'	mRNA	-1.008053645	4.48E-07	uncharacterized LOC412646

408587	'LOC408587'	mRNA	-1.021791808	2.17E-22	histidine decarboxylase
727193	'LOC727193'	mRNA	-1.0230363	1.63E-09	lipase member H-A
413069	'LOC413069'	mRNA	-1.023180082	7.92E-18	H(+)/Cl(-) exchange transporter 7
411807	'LOC411807'	mRNA	-1.043998757	1.37E-15	uncharacterized LOC411807
410915	'LOC410915'	mRNA	-1.044396081	3.03E-36	aquaporin AQPAn.G
726446	'LOC726446'	mRNA	-1.046433614	8.61E-76	uncharacterized LOC726446
409919	'LOC409919'	mRNA	-1.057889438	1.40E-06	excitatory amino acid transporter 1
409078	'LOC409078'	mRNA	-1.092838456	2.74E-08	uncharacterized LOC409078
100578805	'LOC100578805'	mRNA	-1.139725172	4.37E-08	putative malate dehydrogenase 1B
726072	'LOC726072'	mRNA	-1.146496371	6.88E-39	putative defense protein 3
726472	'LOC726472'	mRNA	-1.158022625	2.31E-04	prohormone-3
724762	'LOC724762'	mRNA	-1.175656299	7.54E-12	ras-related protein Rab-28
724433	'LOC724433'	mRNA	-1.197239021	3.28E-04	kell blood group glycoprotein homolog
410654	'LOC410654'	mRNA	-1.210792502	3.30E-106	cholecystokinin receptor-like
411814	'LOC411814'	mRNA	-1.212217584	2.00E-04	kinesin 2C
100126690	'Pban'	mRNA	-1.239059197	7.64E-28	pheromone biosynthesis-activating neuropeptide
107965774	'LOC107965774'	mRNA	-1.241453712	2.48E-11	uncharacterized LOC107965774
410566	'LOC410566'	mRNA	-1.243253843	3.41E-194	putative inorganic phosphate cotransporter
102653795	'LOC102653795'	mRNA	-1.251883237	8.04E-07	neuropeptide Y receptor type 1
100578532	'LOC100578532'	mRNA	-1.285155611	8.80E-04	uncharacterized LOC100578532
100577543	'LOC100577543'	mRNA	-1.288375515	3.66E-21	fez family zinc finger protein 1
413894	'Y-e3'	mRNA	-1.297848528	1.40E-11	yellow-e3
406065	'Wat'	mRNA	-1.330496025	7.83E-58	worker-enriched antennal transcript
412797	'LOC412797'	mRNA	-1.341239591	4.80E-33	facilitated trehalose transporter Tret1
411457	'LOC411457'	mRNA	-1.363520703	6.25E-38	galactosylgalactosylxylosylprotein 3-beta-glucuronosyltransferase P
551579	'LOC551579'	mRNA	-1.382017151	9.79E-24	band 4.1-like protein 5
410515	'LOC410515'	mRNA	-1.383701811	5.00E-38	uncharacterized LOC410515
100578732	'LOC100578732'	mRNA	-1.409899904	2.40E-13	uncharacterized LOC100578732
551582	'LOC551582'	mRNA	-1.436670527	3.12E-26	maternal protein exuperantia

725457	'LOC725457'	mRNA	-1.48032298	1.63E-12	sodium channel protein Nach
726639	'LOC726639'	mRNA	-1.505413961	6.15E-07	oxidized low-density lipoprotein receptor 1
107963975	'LOC107963975'	mRNA	-1.546962918	1.18E-42	uncharacterized LOC107963975
107965323	'LOC107965323'	mRNA	-1.601629276	2.62E-04	uncharacterized LOC107965323
100577932	'LOC100577932'	mRNA	-1.617059317	3.80E-08	uncharacterized LOC100577932
411414	'LOC411414'	mRNA	-1.635576608	2.96E-04	dynein assembly factor with WDR repeat domains 1
412007	'LOC412007'	mRNA	-1.636186498	3.35E-14	facilitated trehalose transporter Tret1
409603	'LOC409603'	mRNA	-1.672551062	5.50E-23	coiled-coil domain-containing protein 142
724565	'LOC724565'	mRNA	-1.688876161	3.38E-70	trypsin-7
725920	'LOC725920'	mRNA	-1.689856459	1.33E-10	vitellogenin receptor
406121	'Mrjp3'	mRNA	-1.69473868	3.66E-06	major royal jelly protein 3
100859932	'LOC100859932'	mRNA	-1.717106493	3.66E-04	uncharacterized LOC100859932
100576886	'LOC100576886'	mRNA	-1.753632369	4.72E-34	uncharacterized LOC100576886
727131	'LOC727131'	mRNA	-1.766688522	4.03E-04	spidroin-2-like
408595	'LOC408595'	mRNA	-1.782201522	6.26E-10	locomotion-related protein Hikaru genki
113219320	'LOC113219320'	mRNA	-1.801144724	6.25E-22	uncharacterized LOC113219320
102656398	'LOC102656398'	mRNA	-1.80516267	9.63E-08	chymotrypsin-like protease CTRL-1
551782	'LOC551782'	mRNA	-1.835219904	7.24E-19	bestrophin-4
406104	'AChE-2'	mRNA	-1.897846115	7.87E-43	acetylcholinesterase 2
100576934	'LOC100576934'	mRNA	-1.923557371	7.91E-04	uncharacterized LOC100576934
726934	'LOC726934'	mRNA	-1.948577785	1.08E-109	PI-PLC X domain-containing protein 1
724867	'LOC724867'	mRNA	-1.970863086	5.00E-04	elongation of very long chain fatty acids protein AAEL008004
102653636	'LOC102653636'	mRNA	-2.016666775	8.26E-04	uncharacterized LOC102653636
726859	'LOC726859'	mRNA	-2.107310864	3.77E-05	farnesyl pyrophosphate synthase-like
410630	'LOC410630'	mRNA	-2.28968527	1.95E-04	cGMP-specific 3',5'-cyclic phosphodiesterase
725157	'LOC725157'	mRNA	-2.33292612	5.53E-33	uncharacterized LOC725157

100577143	'LOC100577143'	mRNA	-2.401330626	5.52E-07	uncharacterized LOC100577143
113218833	'LOC113218833'	mRNA	-2.554323561	4.06E-26	transmembrane protein 234 homolog
102655922	'LOC102655922'	mRNA	-2.889495535	8.94E-48	uncharacterized LOC102655922
102654641	'LOC102654641'	mRNA	-4.942666194	8.54E-36	uncharacterized LOC102654641
